# Cortisone and cortisol break hydrogen-bonding rules to make a drug–prodrug solid solution

**DOI:** 10.1107/S2052252520013263

**Published:** 2020-10-23

**Authors:** Vivek Verma, Simone Bordignon, Michele R. Chierotti, Monica Lestari, Kieran Lyons, Luis Padrela, Kevin M. Ryan, Matteo Lusi

**Affiliations:** a Department of Chemistry and Bernal Institute, University of Limerick, Limerick, Ireland; b Dipartimento di Chimica, Università di Torino, Torino, Italy

**Keywords:** solid solutions, mixed crystals, multidrug formulations, steroids, pharmaceutical solids, assisted spray drying, hydrogen bonding, hydrocortisone, cortisone

## Abstract

Multidrug products enable more effective therapies and simpler administration regimens, provided that a stable formulation is prepared, with the desired composition. In this view, solid solutions have the advantage of combining the stability of a single crystalline phase with the potential of stoichiometry variation of a mixture. Here a drug–prodrug solid solution of cortisone and cortisol (hydrocortisone) is described. Despite the structural differences of the two components, the new phase is obtained both from solution and by supercritical CO_2_ assisted spray drying. In particular, to enter the solid solution, hydrocortisone must violate Etter’s rules for hydrogen bonding. As a result, its dissolution rate is almost doubled.

## Abstract 2

In order to form a solid solution with its prodrug cortisone, hydrocortisone (cortisol) must violate Etter’s rule of hydrogen bonding. The preparation of a uniform product with the desired composition, which is hindered under a thermodynamic regime, can be achieved by supercritical assisted spray drying. The new phase enables fine-tuning of the phase’s composition as well as a higher dissolution rate for hydrocortisone.

## 1. Introduction   

Modern medical therapies rely on increasingly complex pharmaceutical regimens that include multiple drugs with synergistic or complementary effects. In the case of chronic conditions, such therapies may be continued throughout the patient’s life. Multidrug formulations could reduce drug dosage and potential side effects whilst simplifying administration regimens (Aljuffali *et al.*, 2016 ▸; Okuda & Kidoaki, 2012 ▸; Das *et al.*, 2010 ▸). Such products can often be prepared as physical mixtures of the active pharmaceutical ingredients (APIs) in the desired dose, although the resulting multiphase system might be difficult to process and to store over time (Raimi-Abraham *et al.*, 2017 ▸). Alternatively, a stable crystalline phase can be obtained by combining multiple active ingredients in a cocrystal (Kavanagh *et al.*, 2019*a* ▸; Bordignon *et al.*, 2017 ▸). Cocrystals possess the advantages of a single phase but their fixed stoichiometry does not allow for the adjustment of the APIs dose, which is necessarily dictated by therapeutic considerations rather than crystallographic ones (Kavanagh *et al.*, 2019*b* ▸). A third approach can be imagined that involves crystalline molecular solid solutions (Lusi, 2018*a* ▸), which combine the simplicity of a single crystalline phase with the stoichiometry variability of a physical mixture.

Solid solutions are commonly employed in inorganic chemistry and metallurgy, whereas their molecular subgroup remains largely understudied. Besides a few notable exceptions (Mishra *et al.*, 2015 ▸; Braga *et al.*, 2009 ▸; Delori *et al.*, 2014 ▸), the dominant perception around these phases is that they are difficult to make (Lusi, 2018*b* ▸). Indeed, empirical rules originally formulated by Hume-Rothery (1926 ▸) and Kitaigorodsky (1984 ▸) prescribe that only atoms and molecules of the same size, charge and shape can mutually substitute each other in the solid state. Moreover, complete solubility is only deemed possible for those compounds that produce isostructural (and/or isomorphous) crystals (Kitaigorodsky, 1984 ▸). In particular, it was suggested that in order to form a mixed crystal, molecules must have equivalent hydrogen-bond donors and acceptors. From a crystal-engineering perspective, such a requirement can be seen as a direct consequence of Etter’s rule for hydrogen bonds: ‘All good proton donors and acceptors are used in hydrogen bonding’ (Etter, 1990 ▸) – a rule that is generally followed in the known crystal structures with a few exceptions owing to steric hindrance (Wood & Galek, 2010 ▸). Ultimately, only a subset of molecules, often differing by a methyl or a halogen substituent, would fulfil Hume-Rothery and Kitaigorodsky prescriptions. Such conditions represent a bottleneck to the development of pharmaceutical solid solutions (Etter, 1990 ▸).

In contrast, recent work shows that appropriate design strategies can enable mixed crystals despite the lack of isostructurality (Schur *et al.*, 2015 ▸) or large size difference (Lestari & Lusi, 2019 ▸) of the parent components. Other works show that the appropriate synthetic conditions could afford long-lasting metastable products – as an example, solvent-assisted grinding can afford solid solutions that are not available by conventional techniques (Chierotti *et al.*, 2010 ▸). In fact, for non-stoichiometric systems, the complex equilibria between liquid and solid phases often hinders the preparation of a uniform product. In those cases, mechanochemical reactions might afford better control over the product by avoiding a liquid phase (Lusi, 2018*b* ▸). Similarly, the kinetic control possible through rapid expansion of supercritical solutions (RESS) processes affords high miscibility in the solid solutions of anthracene/phenanthrene (Liu & Nagahama, 1996 ▸), l-leucine/l-isoleucine and l-leucine/l-valine (Raza *et al.*, 2018 ▸).

With these premises we believe that the investigation of novel pharmaceutical solid solutions and their potential scale up by such methods is meritable of attention. In particular, we wanted to test whether solid solutions could be formed for molecules that have different hydrogen-bonding capabilities and are not isostructural. To this end, our attention was directed to two steroids: cortisone (C) and hydrocortisone (HC).

Owing to their extensive use in medicine, many steroids are synthesized and commercialized as slow-releasing formulations that help in maintaining the ideal blood concentration for prolonged periods of time (Paik *et al.*, 2019 ▸; Krasselt & Baerwald, 2016 ▸). Over five decades ago, steroids were among the earliest APIs to be co-crystallized as solid solutions and eutectic mixtures (Rudel, 1974 ▸; Castellano *et al.*, 1980 ▸). Interestingly, solid solutions are also reported for pairs of steroids such as arenobufagin/gamabufotalin and cinobufagin/cinobufotalin (Kálmán & Párkány, 1997 ▸), which are ‘essentially isostructural’ despite their different hydrogen-bond capabilities. In fact, for large and non-polar molecules such as steroids, the role of dispersive forces may become predominant over hydrogen bonds (Thompson & Day, 2014 ▸).

C was the first steroid to be employed as a replacement in adrenocortical deficiency states (Benedek, 2011 ▸; Hench *et al.*, 1949 ▸). Nowadays, it is largely substituted by its more soluble metabolite: cortisol (HC), the most widely used steroidal anti-inflammatory drug, listed by the World Health Organization as an essential medicine (Garay *et al.*, 2007 ▸). For this drug, the topical market alone is estimated at ∼3 billion USD globally.

**Figure 1:**
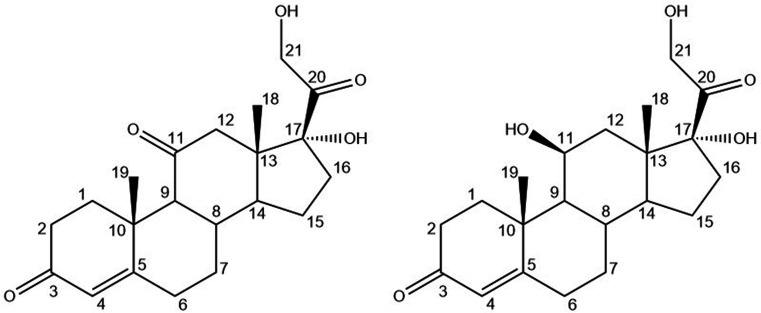
Schematic representations of C (left) and HC (right), with C atom numbering.

C and HC differ only in the substituent in the C11 position, a carbonyl and a hydroxyl moiety, respectively (Fig. 1 ▸), and their metabolism is closely related. In fact, C can be seen as a prodrug (Becker, 2001 ▸) of HC, being converted to the latter by the 11β-hydroxysteroid dehydrogenase (Lakshmi & Monder, 1985 ▸; Edwards *et al.*, 1988 ▸). From a pharmaceutical perspective, it can be imagined that a solid form that includes both molecules could help maintain the desired plasma concentration for a prolonged period reducing the number of doses and simplifying their administration.

## 2. Results and discussion   

### 2.1. Single-crystal analysis   

Despite their similar biochemical functions, C and HC are rather different from a supramolecular point of view. They crystallize in different structures and show different polymorphism. C has only one known polymorph in the *P*2_1_2_1_2_1_ space group [CCDC (Groom *et al.*, 2016 ▸) refcode DHPRTO; Declercq *et al.*, 1972 ▸], while three polymorphs of HC are known: forms I (CCDC refcode ZZZPNG01) and III (CCDC refcode ZZZPNG03) in the *P*2_1_2_1_2_1_ space group and form II (CCDC refcode ZZZPNG02) in the monoclinic *P*2_1_ space group (Suitchmezian *et al.*, 2008 ▸). No evident structural similarity is recognisable in these structures (Kálmán & Párkány, 1997 ▸), phenomena that can be explained in terms of the different hydrogen-bond capabilities of the substituents, carbonyl and hydroxyl, on the C11.

Slow solvent evaporation of alcoholic solutions of C and HC in 2:1, 1:1 and 1:2 ratios provided colourless crystals. Single-crystal X-ray diffraction (XRD) revealed that the crystals are all isomorphous to the C structure (CCDC refcode DHPRTO). The C11–O11 bond lengths for the single crystals isolated from the solutions measure 1.25, 1.29 and 1.31 Å, respectively (Table 1 ▸). These values fall between those of pure C (C = O = 1.21 Å) and those of pure HC (C–O = 1.43 Å) and suggest the formation of a solid solution. The poor resolution of standard XRD does not allow for the refinement of the oxygen substituent as a split atom (carbonyl versus hydroxyl group), nor for the reliable refinement of hydrogen-atoms occupancy (Lusi & Barbour, 2011 ▸). Similarly, the single crystals are too small for high-performance liquid chromatography (HPLC) analysis. The occupancy refinement for the hydrogen atoms on C11 and on the adjacent O11 was attempted for a mere aesthetical end. The single crystals, isolated at the beginning of the crystallization, appear enriched in HC. Incidentally, when the C–O bond lengths are plotted against the calculated occupancy, a second-order relation emerges (see Fig. S1 in the Supporting information).

**Figure 2:**
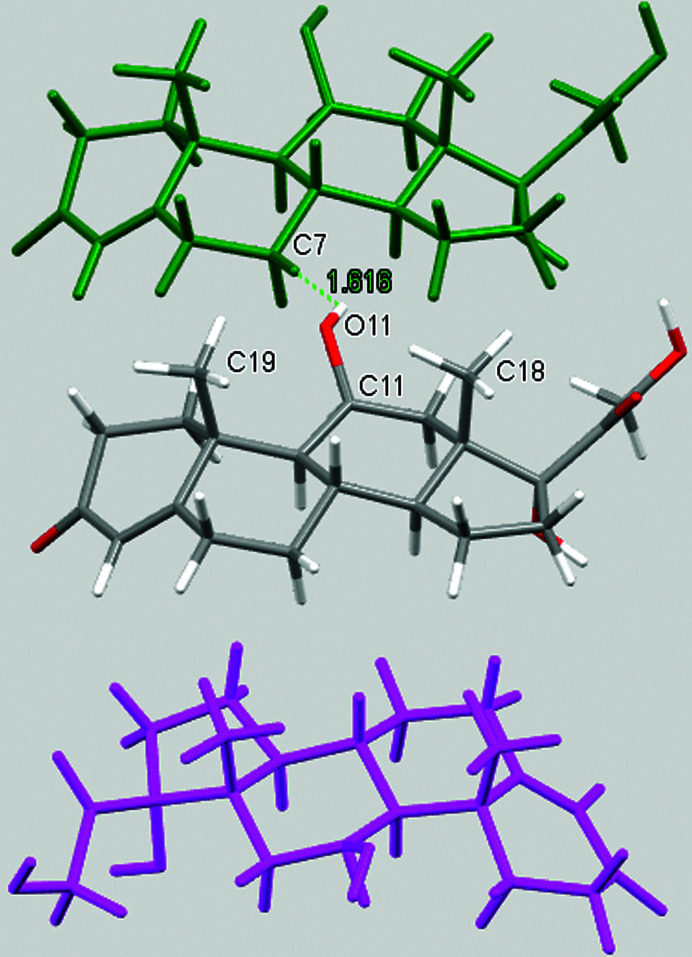
Part of the packing features of HC in the structure of C. The green molecule is generated by translation along *a* and the purple molecule is generated by screw symmetry along *b*.

A qualitative understanding of the structure modifications that occur with substitution can be proposed using Hirshfeld surface analysis. Here the (virtual) replacement of a HC molecule in the structure of C would result in a short H⋯H contact (∼1.8 Å) between the hydroxyl group O11 and C7 in the adjacent molecule (Fig. 2 ▸). This value is ∼20% shorter than the expected contact distance based on the Bondi radius (1.1 Å). At the same time, any rotation of the OH group is sterically hindered by the presence of the adjacent methyl carbons C18 and C19, which suggests that the substitution would rapidly increase the enthalpy of the crystal. As a response, when the amount of HC increases, the structure progressively relaxes to accommodate the bulkier hydroxyl substituent (Table 1 ▸). The structure adjustments have negligible effects on the contact surface of C but the H⋯H contact distance for HC increases to above 2 Å. A similar phenomenon was observed in the phenazine/acridine system, confirming the importance of structure modulability for the successful realization of solid solutions (Schur *et al.*, 2015 ▸).

The correct identification and quantification of all the energy contributions in such a disordered system is not straightforward and it is beyond the scope of this work, though an estimate of the energies can be performed in *Crystal Explorer* (Turner *et al.*, 2017 ▸) for the C and HC molecules in the different structures. The results indicate that the overall interaction energy for the HC molecule in the structure of pure C is ∼12 kJ mol^−1^ higher than that of the C molecule (see Table S1 in the Supporting information). The main difference is caused by the higher repulsion occurring with another HC molecule related by simple translation along the *a* axis (identified as *x*, *y* and *z* in Table S1 and coloured in green in Fig. 2 ▸). Although, part of the repulsion is compensated by a greater dispersive contribution. As the substitution increases and the structure adjusts, the repulsion contribution between this pair of HC molecules is progressively reduced and, eventually, the total interaction energy calculated for HC becomes comparable with the one calculated for C. Notably, the structural and compositional variations along the series seem to have little effect on the other molecular interactions. Invariantly, the largest contribution to the molecular packing comes from the dispersive interaction with the molecule along the *b* screw axis (identified as −*x*, *y* + 1/2, −*z* + 1/2 in Table S1 and coloured in violet in Fig. 2 ▸).

### 2.2. Bulk synthesis and properties   

**Figure 3:**
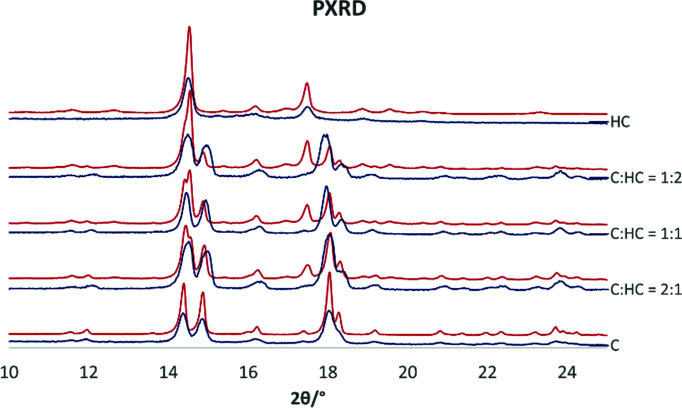
Powder XRD patterns measured for microcrystalline powder generated by the SASD method (blue) and the mechanochemical product (red).

Based on previous experience, solvent assisted co-grinding of the two molecules was attempted to make a bulk product with homogenous 2:1, 1:1 and 1:2 ratio compositions. The persistence of a diffraction peak at 17.5° shows that a phase mixture was obtained (Fig. 3 ▸). Such qualitative conclusions are confirmed by Rietveld refinement, according to which the relative amount of each phase coincides with that of the starting materials (Table S1). On the contrary, the same technique indicates that a single microcrystalline phase is obtained when CO_2_ is employed in a supercritical CO_2_ assisted spray drying (SASD) process (Long *et al.*, 2019 ▸, 2020 ▸; Padrela *et al.*, 2017 ▸) (Fig. 3 ▸). In this case, peak broadening indicates smaller crystallites.

**Figure 4:**
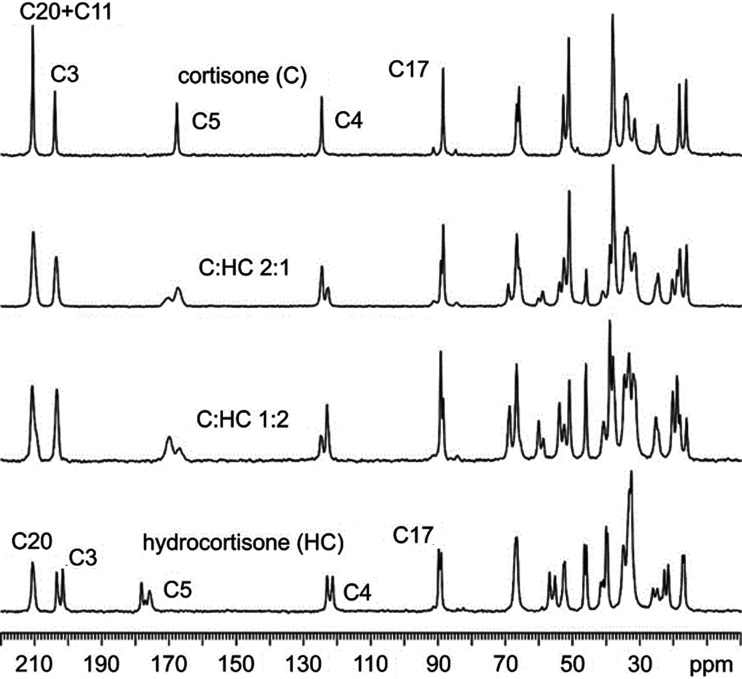
^13^C (100.63 MHz) CPMAS SSNMR spectra measured for the microcrystalline powder generated by the SASD method. The labels refer to assignments of relevant peaks (see Fig. 1 ▸ for atom numbering).

The spray-drying methods ensure that the overall stoichiometry of the microcrystalline product coincides with that of the liquid phase. Within the bulk, product uniformity is confirmed by solid-state nuclear magnetic resonance (SSNMR). ^13^C cross-polarization magic angle spinning (CPMAS) spectra of the solid solutions are consistent with those of pure C, but with apparent differences, especially in the number of signals (Fig. 4 ▸). This agrees with the fact that the structure of pure C is maintained in the solid solutions, with HC as a guest molecule. The homogeneous nature of the microcrystalline product was further confirmed by ^1^H T_1_ relaxation measurements acquired through ^13^C (not shown). Indeed, the ^1^H T_1_ values are the same for each ^13^C signal, indicating active spin diffusion processes, *i.e.* homogeneous domains over a 100 nm scale. The CPMAS technique is intrinsically non-quantitative, since the intensity of each spectral resonance depends both on their T_XH_ (the cross-polarization rate) and their T_1ρ_^H^ (the proton spin-lattice relaxation in the rotating frame). However, the two CH groups in C and HC are the same functional group (namely, the olefinic CH group – C4), in the same chemical environment of two almost identical and rigid molecules (or in any case with very similar mobility) in the same unit cell. Therefore, it is reasonable and safe to think that the two CH groups in the two molecules have almost identical T_XH_ and T_1ρ_^H^ values and the same cross-polarization rate. Thus, ^13^C spectra were used to achieve reliable quantitative information (Anelli *et al.*, 2019 ▸). Specifically, in order to assess the relative amounts of C and HC in the solid solutions, a deconvolution was performed on the signals in the 120–130 p.p.m. range. By considering the resonances to be the result of slightly different contributions, the deconvolution allowed the determination of the C:HC ratio for the SASD products, in accordance with the nominal ones (see Fig. S3).

**Figure 5:**
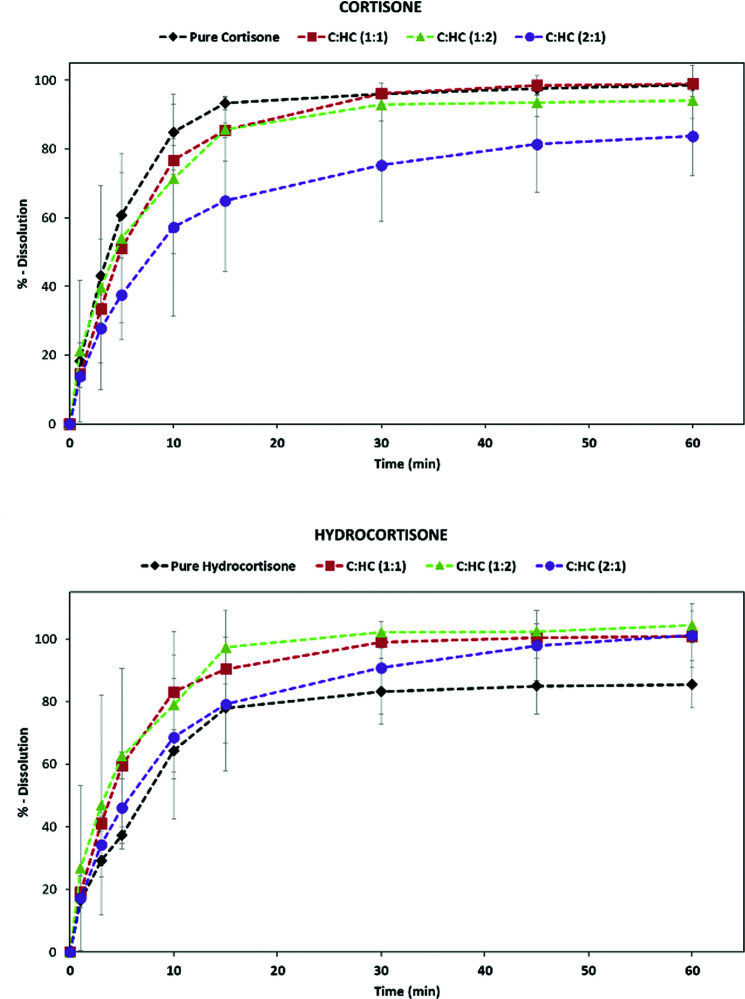
Dissolution profiles of C and HC for the products generated by the SASD method.

Thermal analyses reveal that the solid solutions are marginally lower melting than the pure compounds but remain stable until ∼180 °C (see Figs. S4 and S5). Finally, the solubility of the new phase was assessed by measuring the intrinsic solubility of C and HC in the solid solution and physical mixture. Notably, in the solid solution the initial dissolution rate of HC is twice that of pure HC (form I). On the contrary, the dissolution rate of C is reduced in the solid solution (Fig. 5 ▸). This agrees with the common understanding that the properties of solid solutions often vary regularly between those of the pure components.

## 3. Conclusions   

Drug/prodrug solid solutions of C and HC were prepared in different stoichiometric ratios. The mixed-crystals results are stable in spite of the different hydrogen-bond capabilities of the two molecules and the different crystal structures of their pure phases. We believe that the formation of the solid solution is possible because in large and non-polar molecules the contribution of dispersive forces becomes predominant over that of hydrogen bonds. Moreover, the host structure of C can adjust to accommodate the hydroxyl group of the HC molecule. Interestingly, a uniform polycrystalline phase could be obtained by the SASD method, whereas the solvent evaporation and mechanochemical techniques resulted in a large compositional spread or a mixture of the pure components, respectively.

From a pharmaceutical point of view, the solid solution enables a faster dissolution rate of HC, which is up to twice that measured for the pure compound. We speculate that the higher solubility could increase the bioavailability of HC, while the compresence of C could prolong the desired concentration of the anti-inflammatory API in plasma. The variable ratio of the solid solutions would then allow the optimization of the correct dosage.

Ultimately, this work shows that mixed crystals represent a viable alternative to physical mixtures and cocrystals to formulate multidrug products. In particular, drug–prodrug solid solutions would be particularly targetable because of the structural similarity that is often observed between an active molecule and its biological precursors.

## 4. Experimental   

All reagents grade products were used as purchased without further purification.

### 4.1. Solution synthesis   

Single crystals of pure C and pure HC were recrystallized from a solution of ∼0.2 mmol (72 mg) of the commercial products in ethanol, by slow evaporation in ambient conditions. The mixed crystals of C and HC in the 2:1, 1:1 and 1:2 ratios were obtained in the same conditions from ethanol solutions containing: (*a*) 72.0 mg (0.2 mmol) of C and 36.3 mg (0.1 mmol) of HC, (*b*) 54.1 mg (0.15 mmol) of C and 54.4 mg (0.15 mmol) of HC, and (*c*) 36.1 mg (0.1 mmol) of C and 72.6 mg (0.2 mmol) of HC, respectively. Good-quality single crystals were isolated from the liquid phase as soon as they formed (within two or three days).

### 4.2. Mechanochemical synthesis   

Solid-solution synthesis of C and HC in the 2:1, 1:1 and 1:2 ratios was attempted by manually grinding in an agate mortar: (*a*) 72.0 mg (0.2 mmol) of C and 36.3 mg (0.1 mmol) of HC, (*b*) 54.1 mg (0.15 mmol) of C and 54.4 mg (0.15 mmol) of HC, and (*c*) 36.1 mg (0.1 mmol) of C and 72.6 mg (0.2 mmol) of HC, respectively. In each case, four to five (Pasteur pipette) drops of ethanol were added to the mixture and machination continued until a dry powder was obtained (∼5–10 min).

### 4.3. SASD synthesis   

Solutions of either pure C or HC where prepared by dissolving 150.0 mg of as-received powder in 20 ml of ethanol. Solutions of the mixed steroids in the 2:1, 1:1 and 1:2 ratios were prepared in 20 ml of ethanol by dissolving: (*a*) 100.0 mg (0.28 mmol) of C and 50.0 mg (0.14 mmol) of HC, (*b*) 75.0 mg (0.21 mmol) of C and 75.0 mg (0.21 mmol) of HC, and (*c*) 50.0 mg (0.14 mmol) of C and 100.0 mg (0.28 mmol) of HC, respectively. Full dissolution was completed in an ultrasonic bath (∼15 min). The solutions were then filtered through a 0.2 µm pore-size nylon filter (Whatman Inc., Florham Park, New Jersey). An Agilent Technologies 1260 Infinity II pump was used to pass the solutions at a flow rate of 0.2 ml min^−1^ through a 0.1 cm^3^ high-pressure nozzle equilibrated at 70 °C with heating resistors. In the nozzle, the solutions were mixed with a stream of CO_2_ compressed to 12 MPa using a SFE Process DoseHPP 400-C pump. The supercritical mixture was depressurized in a 1000 cm^3^ chamber in equilibrium with a water jacket at 70 °C, and the product collected on 0.2 µm filter paper. The samples were harvested and stored in a desiccator prior to characterization to prevent exposure to humidity that results in solid-state transformation over time.

### 4.4. XRD analysis   

Single-crystal XRD was performed in Bruker D8 Quest single-crystal X-ray diffractometers with an Mo anode for C, and a Cu anode for HC. Measurements were taken at an ambient temperature. The intensities were integrated with *SHELX* and *SAINT* in the Bruker *APEX3* (Bruker, 2016 ▸) suite of programs and a solution was found using direct methods. Atomic positions and occupancies were refined against all the *F* ^2^_obs_ values, and all non-hydrogen atoms were treated anisotropically in *SHELXL* (Sheldrick, 2015 ▸) using the *X-SEED* (Barbour, 2001 ▸) interface.

Powder XRD patterns were collected on a X’Pert Pro instrument at 40 kV and 40 mA, with Cu *K*α λ = 1.54056 Å in a θ°–θ° geometry. Data were measured from 4 and 40 2θ° with a step size of 0.0167113° and a scan time of 19.685 seconds step^−1^. Samples were placed in a zero-background disc. Rietveld refinement for mixture samples was performed in *HighScore Plus* (Degen *et al.*, 2014 ▸). Rietveld refinement was performed for the mixture samples against the pure C and HC crystal structures.

### 4.5. Hirshfeld surface analysis and energy calculation   

Hirshfeld surface analysis was calculated with *Crystal Explorer 17* (Turner *et al.* 2017 ▸) for the molecules of C and HC in the pure compounds and in the solid solutions. Since the hydrogen-atom position cannot be reliably refined by XRD, the hydrogen atoms were fixed according to the riding model and their bond lengths normalized to the average neutron data. In particular, for the HC molecule, the H-C11-O11-H torsion angle was fixed to 45°: the most common value obtained as a result of the structure refinement. This precaution allows a more direct comparison of the structural change as a function of composition.

Interaction energies were calculated in *Crystal Explorer 17* for a cluster containing the reference molecule and neighbour molecules within a radius of 3.8 Å using the default setting and the HF/3-21G basis set.

### 4.6. Solid-state NMR   

Solid-state NMR spectra were acquired with a Bruker Avance II 400 Ultra Shield instrument, operating at 400.23 and 100.63 MHz for ^1^H and ^13^C nuclei, respectively

The powder samples were packed into cylindrical zirconia rotors with a 4 mm outer diameter and an 80 µl volume. A certain amount of sample was collected from each batch and used without further preparations to fill the rotor. ^13^C CPMAS spectra were acquired at room temperature at a spinning speed of 12 kHz, using a ramp cross-polarization pulse sequence with a 90° ^1^H pulse of 3.6 µs, a contact time of 3 ms, optimized recycle delays ranging from 2.5–5.6 s and a number of scans in the range 200–6500, depending on the sample. For every spectrum, a two-pulse phase modulation decoupling scheme was used, with a radiofrequency field of 69.4 kHz. The ^13^C chemical shift scale was calibrated through the methylenic signal of external standard glycine (at 43.7 p.p.m.). As for the ^13^C T_1_-^1^H analysis of the 1:1 solid solution, ^13^C spectra were acquired for 320 scans with different relaxation delays, included in the range 0.2–60 s and calculated by Bruker *TopSpin* 2.1 software through an exponential algorithm.

In order to assess the relative amounts of C and HC in the solid solutions, a deconvolution was performed on the signals in the 120–130 p.p.m. range (Fig. 2 ▸). By considering the resonances to be the result of slightly different contributions, the deconvolution allowed us to determine that the C:HC ratio for the SASD products is equal to 66.0:34.0, 49.7:50.3 and 35.2:64.8, in accordance with the nominal ones (2:1, 1:1 and 1:2, respectively).

### 4.7. Thermal analysis   

Thermogravimetric analysis was performed on a TA Instrument Q50 with aluminium sample pans. Samples were heated to 400 °C with a rate of 10 °C min^−1^ and nitrogen gas flow rates of 60 ml min^−1^. Differential scanning calorimetry was performed on a TA Instrument Q2000 in sealed aluminium pans with a heating rate of 10 °C min^−1^ and nitrogen gas flow rates of 60 ml min^−1^.

### 4.8. Solubility measurements   

Intrinsic solubility measurements were performed in sink conditions. 20 mg of each sample was dissolved in 1000 ml of deionized water at 37 °C at 150 rev min^−1^. The sample was collected at minutes 1, 3, 5, 10, 15, 30, 45 and 60 and filtered through a 0.2 µm nylon filter. The HPLC system used was Agilent Technologies 1260 Infinity with column Macherey-Nagel EC100/4.6 Nucleodur 100-5C18ec. The mobile phase used was methanol and water with a ratio of 1:1 at 1.0 ml min^−1^ flow rate. 20 µl of the sample was injected into the HPLC system. The system and the autosampler were at an ambient temperature. Chromatograms were recorded at 248 nm with a run time of 12 min. The processing of the chromatographic data was carried out in the software *Chemstation* for liquid chromatography (Agilent Technologies).

## Supplementary Material

Crystal structure: contains datablock(s) C_KL1_C, KL4, C_KL3, KL5, C_KL2_HC. DOI: 10.1107/S2052252520013263/lq5036sup1.cif

Supporting information. DOI: 10.1107/S2052252520013263/lq5036sup2.pdf

CCDC references: 2010415, 2010416, 2010417, 2010418, 2010419

## Figures and Tables

**Table 1:** Crystallographic data for single crystals of C, HC and their solid solutions

	C	C:HC ≃ 2:1	C:HC ≃ 1:2	C:HC ≃ 1:3	HC form I
*a* (Å)	7.7819 (4)	7.7442 (6)	7.7308 (7)	7.76953 (9)	10.1439 (14)
*b* (Å)	10.0468 (5)	10.0968 (8)	10.1237 (9)	10.1258 (11)	12.4255 (16)
*c* (Å)	23.6401 (13)	23.6750 (19)	23.694 (2)	23.694 (3)	30.496 (5)
Volume (Å^3^)	1848.26 (17)	1851.2 (3)	1854.4 (3)	1854.4 (3)	3843.8 (10)
Space group	*P*2_1_2_1_2_1_	*P*2_1_2_1_2_1_	*P*2_1_2_1_2_1_	*P*2_1_2_1_2_1_	*P*2_1_2_1_2_1_
Moiety formula	C_21_H_28_O_5_	0.63(C_21_H_28_O_5_) 0.37(C_21_H_30_O_5_)	0.36(C_21_H_28_O_5_) 0.64(C_21_H_30_O_5_)	0.26(C_21_H_28_O_5_) 0.74(C_21_H_30_O_5_)	C_21_H_30_O_5_
*M*_r_	360.43	361.18	361.71	361.92	359.42
*D*_x_ (g cm^−3^)	1.295	1.296	1.296	1.306	1.242
*Z*	4	4	4	4	8
*R* (reflections)	0.0504 (3395)	0.0747 (2479)	0.0715 (1824)	0.0715 (1824)	0.0969 (931)
Temperature (K)	296	296	296	296	296
C–O distance (Å)	1.208	1.255	1.288	1.311	1.457
Hirshfeld plot of cortisone	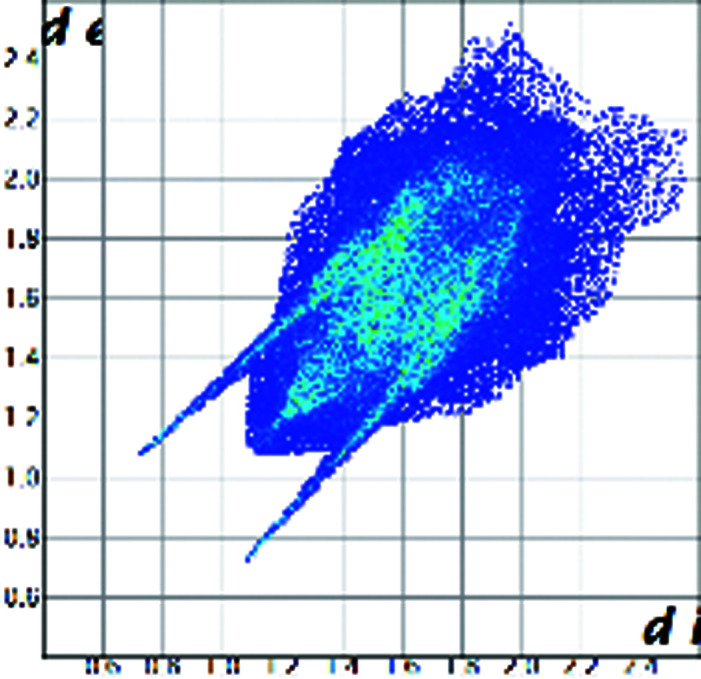 [Chem scheme1]	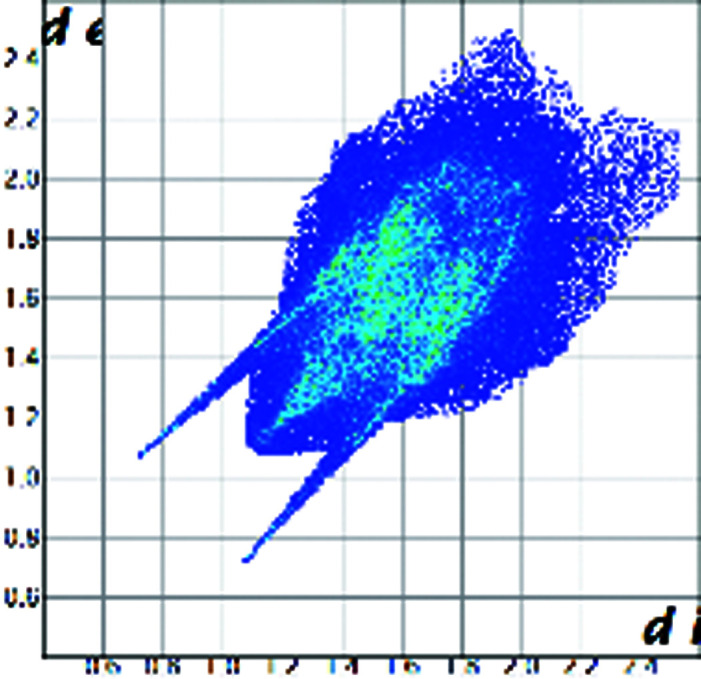 [Chem scheme2]	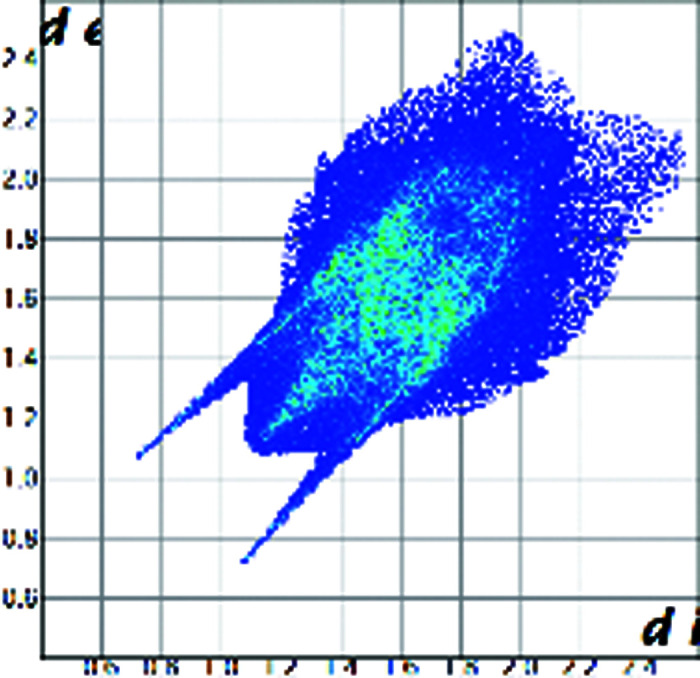 [Chem scheme3]	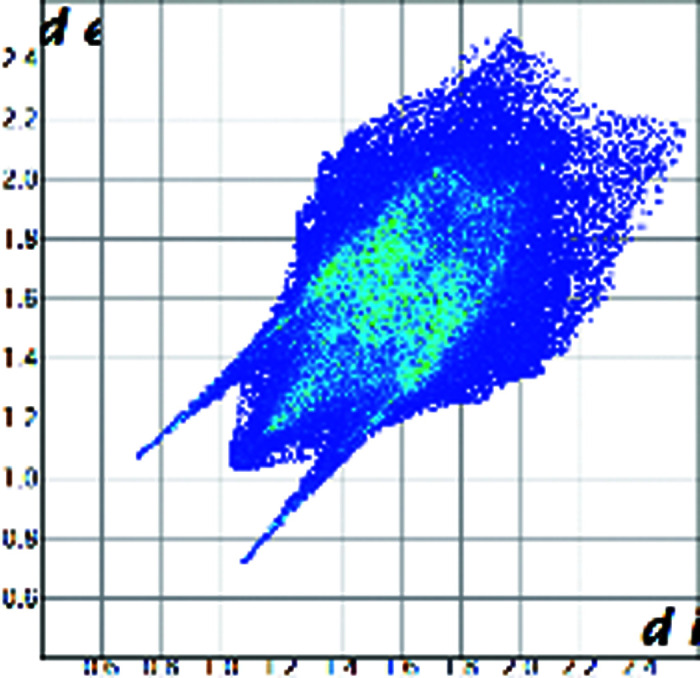 [Chem scheme4]	
Hirshfeld plot of hydrocortisone	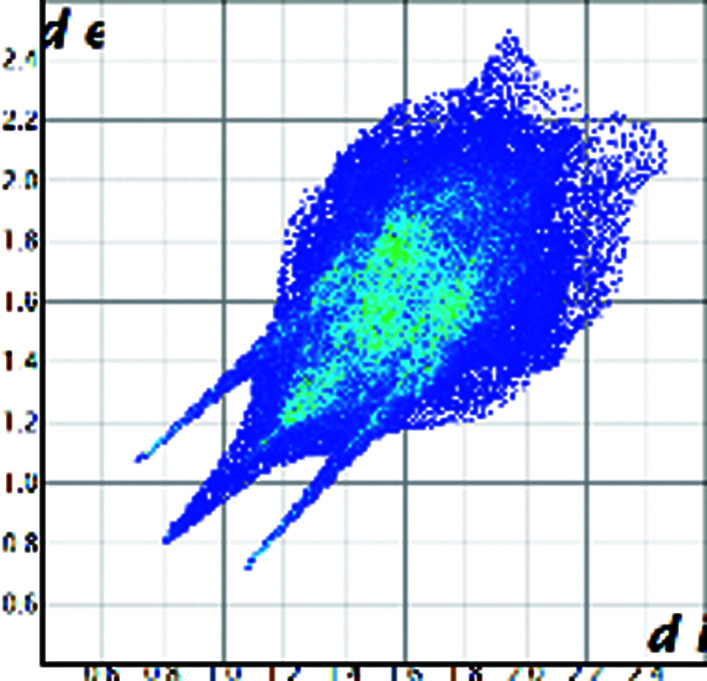 [Chem scheme5]	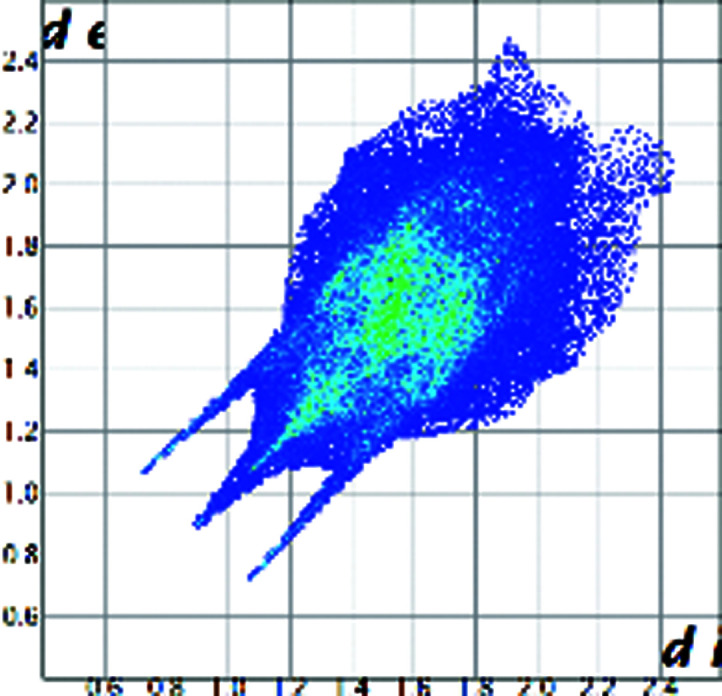 [Chem scheme6]	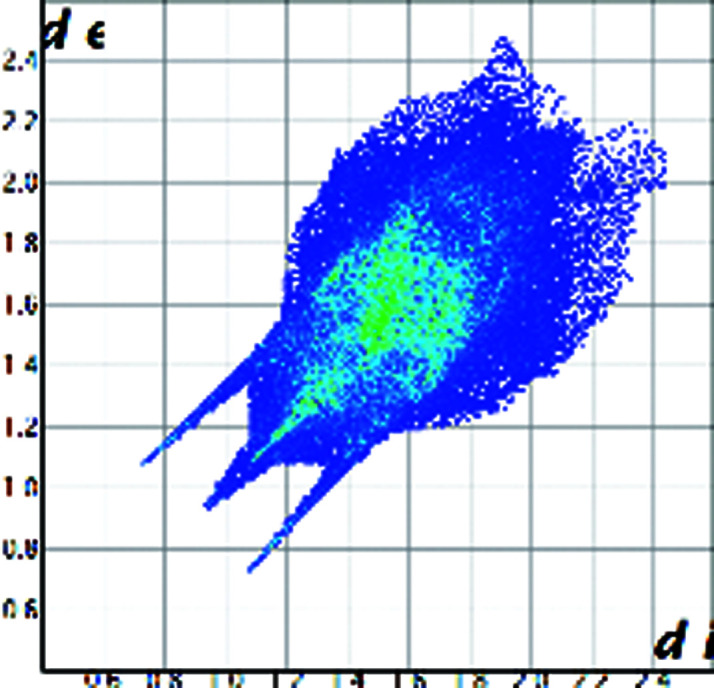 [Chem scheme7]	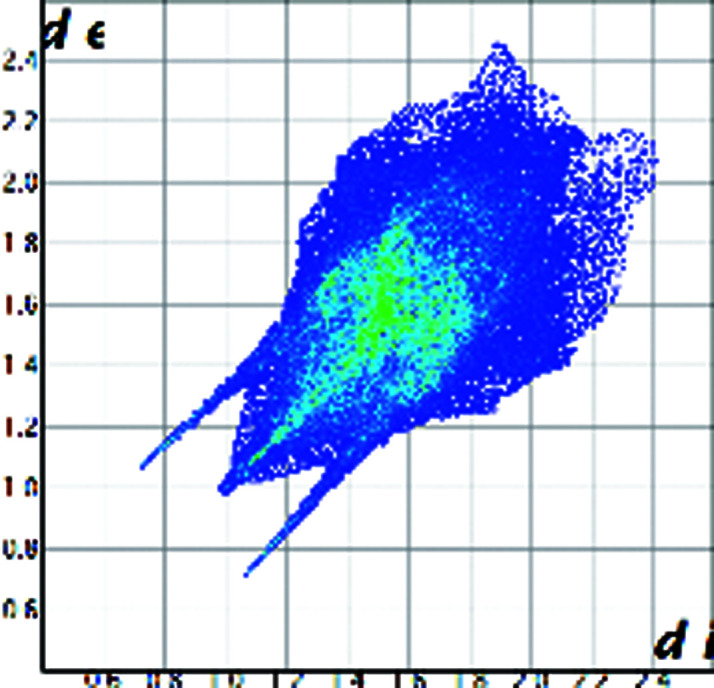 [Chem scheme8]	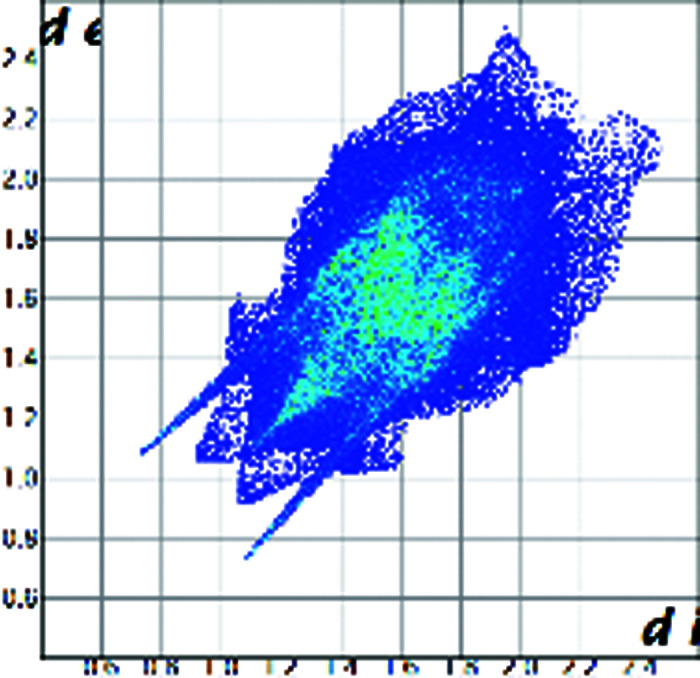 [Chem scheme9]
