# Supplementary material for: Cortisone and cortisol break hydrogen-bonding rules to make a drug–prodrug solid solution
Source: IUCrJ. 2020 Oct 23;7(Pt 6):1124–30. doi: 10.1107/S2052252520013263 (PMC7642785; doi:10.1107/S2052252520013263)
Supplement: Supplementary file 2 [file m-07-01124-sup2.pdf]

IUCrJ

Volume 8 (2021)

Supporting information for article:

Cortisone and cortisol break hydrogen-bonding rules to make a drug-prodrug solid solution

Vivek Verma, Simone Bordignon, Michele R. Chierotti, Monica Lestari, Keiran Lyons, Luis Padrela, Kevin M. Ryan and Matteo Lusi

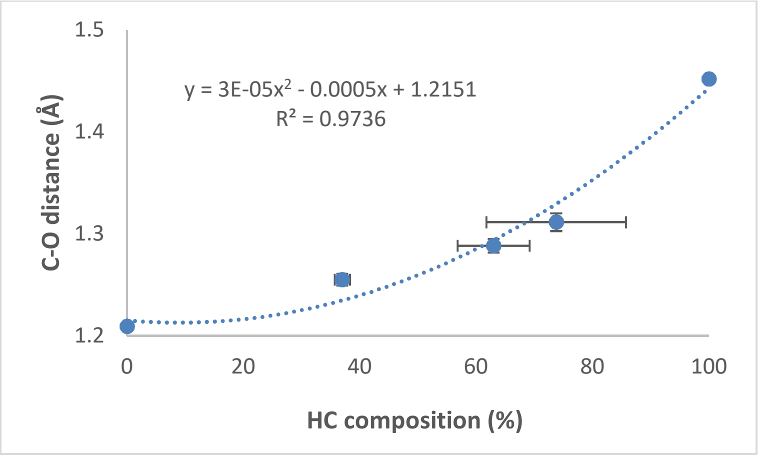

Figure S1 Plot of C-O distance vs nominal composition measured from single crystal XRD data.

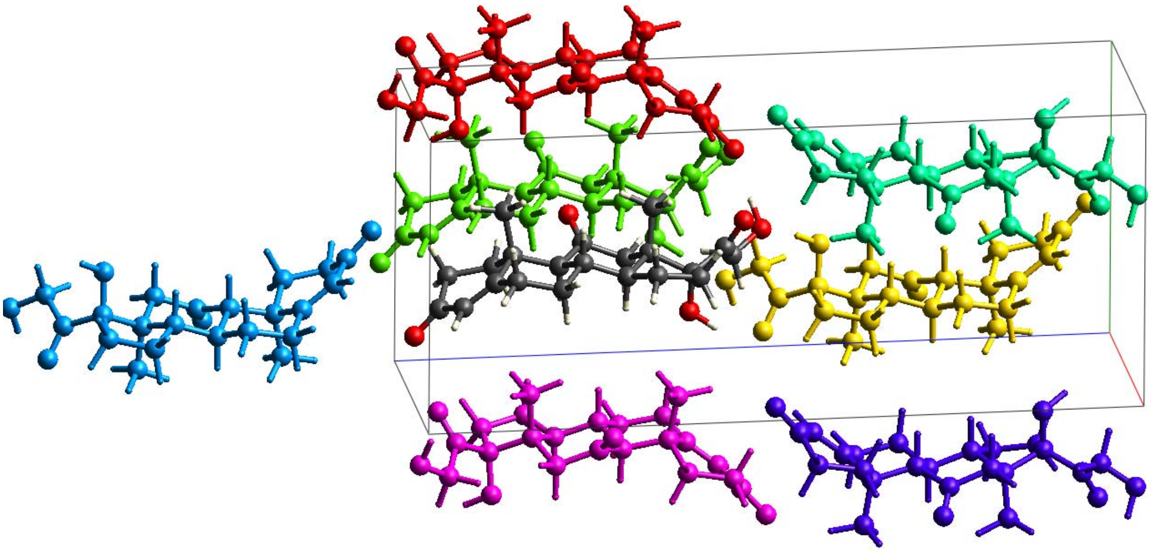

Figure S2 Cortisone crystal packing; the molecular colour matches the one in Table S1.

Table S1 Interaction energies for cortisone (left) and hydrocortisone (right) in the solid solutions.

C in pure C	Symop	R	E_ele	E_pol	E_dis	E_rep	E_tot
	-x, y+1/2, -z+1/2	6.46	-43.9	-12.4	-40.1	44	-53.3
	x+1/2, -y+1/2, -z	11.9	-51.4	-16.8	-17.3	37.7	-48.3
	x, y, z	7.78	-2.4	-2.8	-25.2	10.1	-18.8
	-x+1/2, -y, z+1/2	12.11	-6.4	-4.1	-17	5.3	-20.2
	x+1/2, -y+1/2, -z	13.4	-5.4	-3.4	-11.7	3.9	-15.1
	-x+1/2, -y, z+1/2	13.96	-9.9	-1.1	-1.7	0	-12.3
	-x, y+1/2, -z+1/2	6.35	-5.4	-5	-53.1	21	-39.6
							-207.6
**C in C:HC = 2:1**	-x, y+1/2, -z+1/2	6.48	-34	-10.1	-39.9	34.3	-49.3
	x+1/2, -y+1/2, -z	12	-53.7	-17.4	-17	37.1	-51.2
	x, y, z	7.73	-1.3	-2.1	-26.2	9.2	-18.9
	-x+1/2, -y, z+1/2	12.1	-8.1	-3.2	-17.5	5.9	-21.4
	x+1/2, -y+1/2, -z	13.4	-6.1	-3.5	-12.1	4.2	-16.1
	-x+1/2, -y, z+1/2	14	-9.9	-1.1	-1.6	0	-12.2
	-x, y+1/2, -z+1/2	6.36	-5.6	-5.1	-51.6	19.7	-39.5
							-208.6
**C in C:HC = 1:2**	-x, y+1/2, -z+1/2	6.47	-36.9	-10.7	-40.3	37.2	-50.6
	x+1/2, -y+1/2, -z	11.9	-53.6	-17.4	-17.4	37.3	-51.3
	x, y, z	7.7	-1	-2.1	-27.2	9.8	-19
	-x+1/2, -y, z+1/2	12.1	-8	-3.5	-17.5	5.9	-21.4
	x+1/2, -y+1/2, -z	13.4	-5.8	-3.4	-12.4	4.4	-15.8
	-x+1/2, -y, z+1/2	14	-9.7	-1	-1.6	0	-12
	-x, y+1/2, -z+1/2	6.34	-5.5	-5	-52.1	21.5	-38.4
							-208.5
**C in C:HC = 1:3**	-x, y+1/2, -z+1/2	6.47	-36.8	-10.7	-40	37	-50.5
	x+1/2, -y+1/2, -z	12	-53.4	-17.3	-17.1	37.4	-50.8
	x, y, z	7.74	-1.5	-2.2	-25.8	9.1	-18.9

HC in pure C	R	E_ele	E_pol	E_dis	E_rep	E_tot
	6.44	-41.5	-13.3	-43.2	43.9	-54.3
	11.93	-51.7	-16.3	-17.3	37.5	-48.4
	7.78	2.6	-7.1	-30.8	27.4	-7.5
	12.11	-4.5	-4.3	-17	5.2	-18.5
	13.42	-5.3	-3.5	-11.8	3.9	-15.1
	13.96	-9	-1.1	-1.7	0	-11.3
	6.37	-6.8	-3.7	-53.2	20.4	-40.7
						-195.8
**HC in C:HC = 2:1**	6.46	-37.1	-12.2	-43.3	40.3	-52.1
	11.95	-52.4	-16.4	-17	36.2	-49.9
	7.74	3.8	-5.5	-31.2	17.5	-13.7
	12.13	-5.6	-3.9	-17.2	5.5	-19.3
	13.43	-5.6	-3.6	-11.9	4	-15.5
	14	-8.8	-1	-1.6	0	-11.1
	6.37	-6.7	-3.7	-52.4	19.9	-40.3
						-201.9
**HC in C:HC = 1:2**	6.47	-35	-11.7	-43.4	38.3	-51.2
	11.96	-52.9	-16.5	-16.9	36	-50.7
	7.73	4.1	-5.1	-31.8	15.8	-15.1
	12.14	-6.2	-3.9	-17.4	5.9	-19.8
	13.44	-5.9	-3.7	-12	4.1	-15.9
	14.02	-8.9	-1.1	-1.6	0	-11.2
	6.37	-6.6	-3.8	-52.1	20	-40
						-203.9
**HC in C:HC = 1:3**	6.46	-35.7	-11.9	-44.4	41	-50.9
	11.93	-54.3	-17	-17.3	37.2	-51.7
	7.7	3.9	-4.8	-33	14.5	-17.2

	-x+1/2, -y, z+1/2	12.1	-7.5	-3.4	-17.2	5.5	-21
	x+1/2, -y+1/2, -z	13.4	-5.8	-3.4	-11.9	4	-15.6
	-x+1/2, -y, z+1/2	14	-9.8	-1.1	-1.6	0	-12.1
	-x, y+1/2, -z+1/2	6.36	-5.4	-5	-52.1	20.2	-39.3

-208.2

	12.1	-5.6	-4	-17.3	5.7	-19.4
	13.39	-5.8	-3.6	-12.5	4.5	-15.8
	13.98	-8.7	-1	-1.7	0	-11
	6.36	-6.6	-3.7	-52.4	21.2	-39.2

-205.2

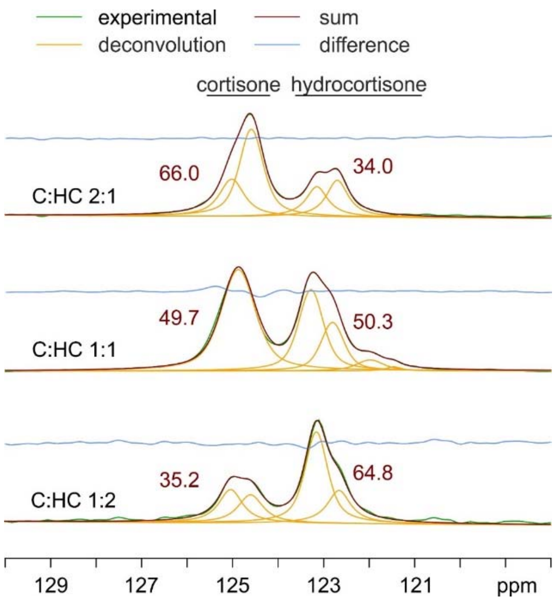

Figure S3 Detail of the ^{13}C CPMAS spectra of the three mixtures (in green), with deconvolved peaks (in yellow). The red line corresponds to the sum of the deconvolved resonances, while the blue line represents the difference between the experimental and the sum spectra. Red labels represent relative amounts (expressed as percent values) of C and HC in the three samples, as calculated through the integral areas of the deconvolved peaks.

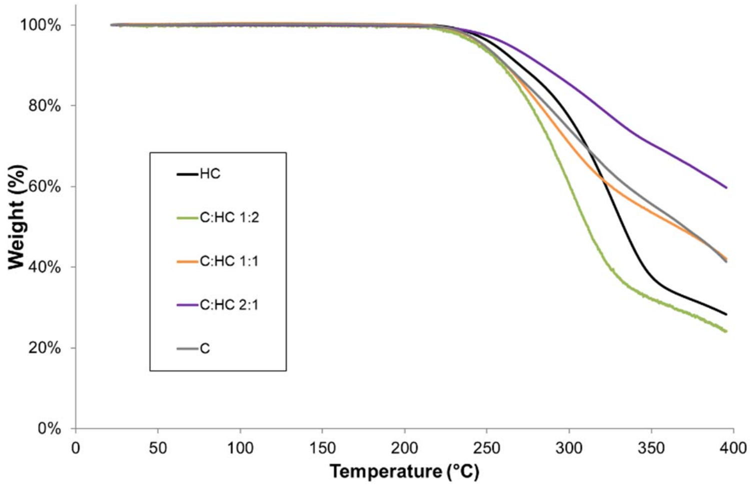

Figure S4 Thermograms measured for pure cortisone (C), pure hydrocortisone (HC) and three C:HC mixed phases generate by SADS (stoichiometric ratios = 2:1; 1:1; 1:2).

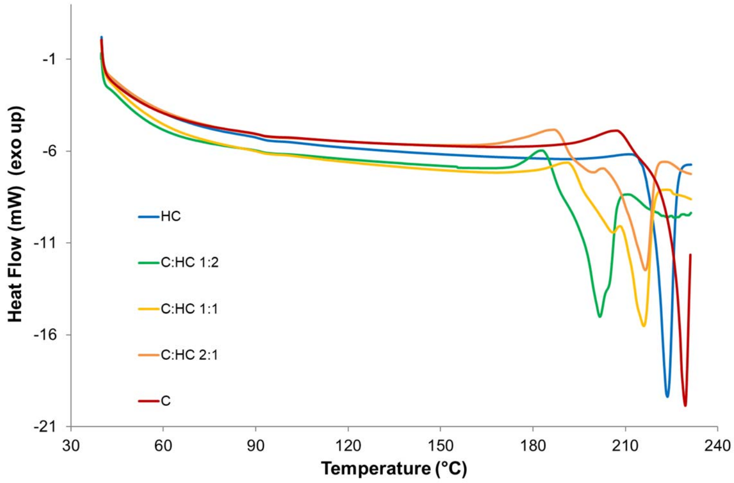

Figure S5 Calorimetric measurements for pure cortisone (C), pure hydrocortisone (HC) and three C:HC mixed phases generate by SADS (stoichiometric ratios = 2:1; 1:1; 1:2).

Table S2 Summary of Phase quantification by Rietveld refinement for the products of mechanochemical (MC) and SADS synthesis.

Sample Ratio		Crystal Form Calculated Weight %		Crystal Form Found (MC synthesis) Weight fraction %		Crystal Form Found (SADS synthesis) Weight fraction %	
**C**	**HC**	**C**	**HC**	**DHPRTO (C)**	**ZZZPNG01 (HC)**	**DHPRTO (C)**	**ZZZPNG01 (HC)**
1	0	100	0	100	0	100	0
1	2	33.18	66.82	32.2(5)	67.8(3)	100	0
1	1	49.86	50.14	48.7(4)	51.3(3)	100	0
2	1	66.58	33.42	65.4(3)	34.6(2)	100	0
0	1	0	100	0	100	0	100
